# Assessing the need for pioglitazone in the treatment of patients with type 2 diabetes: a meta-analysis of its risks and benefits from prospective trials

**DOI:** 10.1038/s41598-020-72967-8

**Published:** 2020-09-25

**Authors:** Binayak Sinha, Samit Ghosal

**Affiliations:** 1 AMRI Hospitals KB 24, KB Block, Sector III, Bidhannagar, Kolkata, 700098 India; 2 Nightingale Hospital, 11 Shakespeare Sarani, Kolkata, 700071 India

**Keywords:** Diabetes, Epidemiology

## Abstract

The safety and usefulness of pioglitazone (Pio) is repeatedly called into question due to the contradictory information available about it. A meta-analysis and risk benefit assessment was conducted to address the various points of debate regarding Pio. Electronic database search (Cochrane library, Embase & PubMed) resulted in 10 citations eligible for this meta-analysis (prospective, randomised studies), which was conducted using CMA software version 3 (Biostat Inc., Englewood, NJ, USA). The meta-analysis was registered with PROSPERO (ID: CRD42019122403) and compared pioglitazone with a control (anti-hyperglycemic agents without pioglitazone) in patients with either established cardiovascular disease or having high cardiovascular risk. Sensitivity and subgroup analysis were conducted to differentiate the effect of Pio against active controls and placebo. The use of Pio compared to the control group that did not use Pio resulted in a 14% and 23% significant reduction in odds of major adverse cardiac events (MACE: MH-OR, 0.86; 95% CI 0.75–0.98), and stroke (MH-OR, 0.77; 95% CI 0.60–0.99), respectively. This reduction in stroke was not significant in comparison to placebo on subgroup analysis. However, Pio significantly increased odds of heart failure (HF) (MH-OR, 1.47; 95% CI 1.26–1.71) as well as hospitalization for heart failure (hHF) (MH-OR, 1.48; 95% CI 1.21–1.81). In addition, the use of Pio was associated with a significant increase in odds of fractures in women (MH-OR, 2.05; 95% CI 1.28–3.27) and anaemia (MH-OR, 2.56; 95% CI 1.55–4.21). Pio has no significant effect on bladder cancer nor macular oedema. Pio has salutary effects on MACE. The positive effects are completely offset by the harm they seem to cause by way of heart failure, fractures, and anaemia. Pio should therefore be reserved for treatment of T2D with high CV risk or established cardiovascular (CV) disease only in selected patients where other antidiabetics are precluded and not routinely.

## Introduction

For years now, the balance between the safety and efficacy of pioglitazone (Pio), a peroxisome proliferator-activated receptor gamma (PPAR γ) agonist, in the treatment of patients with type 2 diabetes (T2D) has been heavily debated. Some experts have even argued that there is minimal role of this drug in treating T2D^1^.

PPAR γ agonists have heterogeneous cardiovascular effects^2^, with rosiglitazone, showing deleterious effects on the cardiovascular system, and these effects translate into a higher rate of myocardial infarction and all-cause mortality associated with its use^3^. In contrast, the use of Pio is associated with not only an improvement of glycaemia, but also an additional reduction of inflammatory markers like high-sensitivity C reactive protein (hs CRP), interleukin 6 (IL 6), and tumor necrosis factor alpha (TNF α) along with a suppression of adhesion molecules like soluble intercellular adhesion molecule (s I CAM), soluble vascular cell adhesion molecule-1 (s VCAM 1), and soluble sE-selectin, indicating improvement of endothelial function^4^. Pio has also been shown to reduce blood pressure^5^ and is associated with improvement of lipid abnormalities^6^ seen in T2D. Surrogate markers of atherosclerotic cardiovascular disease are reduced with Pio when compared to sulphonylureas^7^. These provide indirect evidence of the positive role of Pio in cardiovascular disease seen in T2D. On the other hand, along with these salutary effects, usage of Pio is associated with weight gain and oedema, which would intuitively have negative effects on cardiovascular outcomes. Indeed, there is a large body of evidence demonstrating an increased risk of heart failure with the use of Pio^7, 8^. Pio has also been implicated in fractures, anemia, and macular oedema, along with bladder cancer, creating much discourse about its continued usage^9, 10^.

The doubts have somewhat mellowed with the publication of a few trials highlighting its cardiovascular (CV) benefits^11–17^ including reduction of stroke in pre-diabetes and insulin resistance^18^.

However, with the persistent shadow of doubt on this molecule appearing from time to time, the risk–benefit assessment of this drug should be conducted objectively and scientifically. Previous systematic reviews have addressed only stroke as an outcome or have addressed observational studies only^19, 20^. The most detailed systematic review and meta-analysis on Pio and CV outcomes published in 2016 addressed cardiovascular outcomes in diabetes, pre-diabetes, and insulin resistance and not T2D specifically^21^. Another meta-analysis conducted in 2007 analyzed a spectrum of extremely heterogenous studies and obviously does not include multiple important citations which have been published since^22^.

Thus, a meta-analysis of the CV outcomes of Pio was conducted, addressing only randomized controlled trials (no observational studies were included) in T2D only and not in patients with pre-diabetes and insulin resistance, as had been done earlier, addressing end points as mandated by the FDA guidance of 2008 for assessing cardiovascular outcomes in anti-diabetics, which includes major adverse cardiovascular events (MACE), non-fatal myocardial infarction (MI), non-fatal stroke and hospitalization for heart failure (hHF)^23^. In addition, the adverse effects of Pio were analyzed in detail. The number needed to treat (NNT) and number needed to harm (NNH) were also calculated to illustrate the risk and benefits of treatment with Pio, respectively.

## Methods

This meta-analysis was conducted according to the recommendations of the PRISMA statement^24^ and registered with PROSPERO (ID: CRD42019122403).

### Search strategy

The randomized prospective studies were identified through a thorough database search (Cochrane Library, PubMed, and Embase), which included the MeSH terms “type 2 diabetes”, “thiazolidinedione”, and “Pioglitazone.” Outcome measures were also searched: “major adverse cardiac events,” “MACE,” “myocardial infarction,” “stroke,” “cardiovascular death,” “all-cause mortality,” “heart failure,” “hospitalization for heart failure,” and “microvascular outcomes.” Furthermore, the primary search filters included human data and clinical trials, although no search restrictions on time or language were used. While performing the Cochrane library search, the outcome keywords were clubbed using the Boolean OR. Similarly, pioglitazone, thiazolidinedione, and type 2 diabetes were clubbed using the Boolean OR. The search results were then combined using the Boolean AND to yield the first set of citations. (Supplementary Fig. 1) The initial search was followed up by a detailed manual search filtering the duplicates and selecting those that met the predetermined inclusion criteria. Any citation that compared pioglitazone versus a non-pioglitazone control arm was included for analysis.

### Data extraction

Both authors independently conducted a web-based search for relevant citations dependent on the selected keywords. After identifying the citation from the web-based search, relevant data was extracted using the trial name, surname of the first author, year of publication, study population, place of origin of the study, design of the study, mean age, gender distribution, drugs in the intervention and control groups, dosages of agents in each group, background status related to cardiovascular disease, and duration of follow up. On identification of the basic database to work upon, further data extraction including the identification of MACE, myocardial infarction, stroke, CV death, all-cause mortality, heart failure, hHF, microvascular complications and all adverse events of interest was instituted. Data on anemia, macular edema, fractures, drug discontinuation, heart failure were collected post hoc since these citations were not primarily designed to investigate these parameters. Additional filters included were, a cap on age above 18 years and clinical trials. No restrictions were placed based on language or date of publication. Any disagreements were resolved by conducting additional independent searches on a different day. After the initial process, a manual search was conducted jointly to identify the citations that met the inclusion criteria:

1. Randomized prospective trials on T2D
2. No cap on the number of patients recruited
3. Minimum duration of follow-up: 6 months
4. Reporting of the standardized outcome (macrovascular and microvascular) end points
5. The control group included standard of care without thiazolidinedione (placebo or active control). The other baseline metabolic as well as CV risk parameters should also be matched.

**Figure 1:**
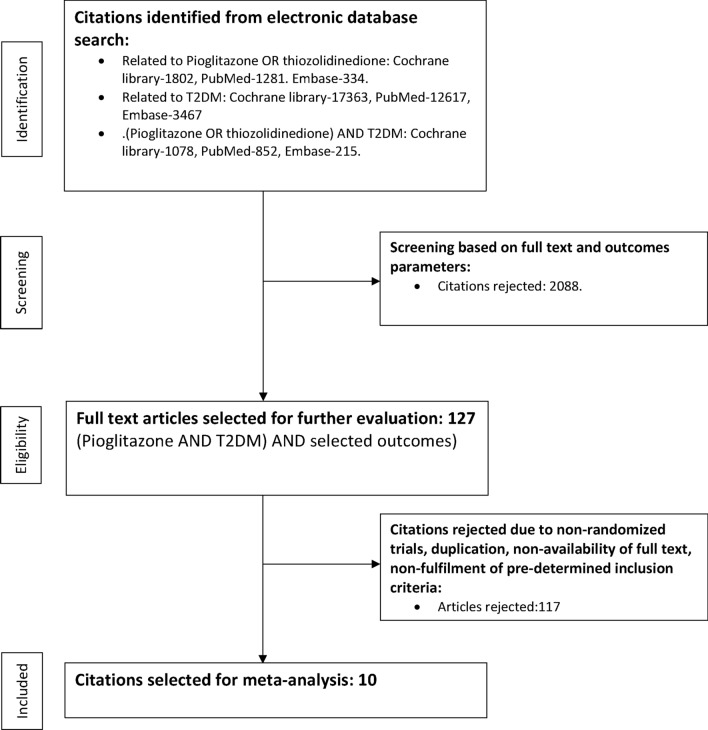
Study selection process.

The process of data extraction is detailed in Fig. 1.

### Quality assessment

The Cochrane risk of bias algorithm was used to assess quality of the studies. The assessment of the individual component of the Cochrane risk of bias algorithm was based on the attributes of those parameters detailed by Higgins and Altman^25^. The authors (based on mutual consensus) after reviewing the materials and methods section of all the selected citations, agreed that five studies scored an unclear risk of selection bias because of insufficient information on random sequence generation and allocation concealment. Other studies showed bias due to unclear risk of blinding of participants and outcome assessment as well as selective reporting. An additional web-based search was conducted to locate the original published protocol of the citations included in the analysis. Comparing the intended outcomes to be reported to the ones finally reported helped in identifying selective reporting and other biases, namely biases related to non-declaration of funding and conflict of interests, possibility of baseline imbalances which is difficult to decipher due to absence of publication of the trial protocol prior to conducting the trial, the degree of differences in the imbalances between the two comparative groups, and a pre-adjudicated and pre-specified hierarchical testing protocol.

### Data synthesis and analysis

A detailed and up-to-date analysis from randomized prospective trials was conducted to assess the positive and negative effects of Pio. Since none of the reported adverse events were selected as pre-adjudicated primary or secondary end points, we limited our search to a broad range of outcome trials and examined in detail the primary sources, supplementary materials, and subsequent publications as a sequel to the original one as the principal source of data. Seven of the selected ten citations included patients with established cardiovascular disease (MI, stroke, and HF)^7, 11, 15–17, 26, 27^. The remaining three citations included patients with either a mixed population (established CVD or those with high CV risk)^12^ or at a high risk for CVD^13, 14^. There seemed to be an apparent discrepancy between the studies as far as the control arm was concerned. This was due to the control arm including either placebo^11, 16^, active control^7, 12^, or standard of care without thiazolidinedione^13–15, 17, 26, 27^. However, in effect, the control arm was universally represented by any oral anti-hyperglycemic agent without thiazolidinedione and was therefore uniform in nature. The primary aim of all the studies included macrovascular outcomes, while the secondary endpoints included microvascular benefits and adverse events. The outcomes selected for analysis were MACE, stroke, myocardial infarction (MI), CV death, all-cause mortality (ACM), nephropathy progression, heart failure (HF), hospitalization for heart failure (hHF), overall and bladder cancer, macular edema, anemia, fracture risk in the overall population and in females, and drug discontinuation. The findings were reported as the Mantel–Haenszel odds ratio (MH-OR) with 95% confidence interval, using the Comprehensive Meta-analysis software version 3 (Biostats Inc., Englewood, NJ, USA). Moreover, the fixed or random effect model was used to calculate the effect size estimation with MH-OR (both individual studies as well as the final effect size) depending on the heterogeneity, which was assessed using Cochran’s Q and Higgins’s I^2^ tests (an I^2^ ≥ 75% indicating considerable heterogeneity), and study characteristics (gross differences in estimated effect size as evident from differences in baseline characteristics). The magnitude of clinical benefit or harm was quantified using the inverse of absolute risk reduction, known as the number needed to treat (NNT). In case of adverse effects, the NNH was calculated.

In view of the control arm including both active controls (other oral anti diabetic agents) as well as non-active controls (placebo), there is a risk of over-estimating the effect size of the outcome measures of interest. To minimize this bias, a subgroup and sensitivity analysis was undertaken by segregating the active-control and the placebo groups (subgroups) and analyzing the selected outcome measures using the odds ratio (sensitivity analysis).

## Results

The meta-analysis was performed on a pooled population of 10,890 patients from ten citations, with 5452 individuals on pioglitazone and 5525 individuals in the control group. The Cochrane risk of bias algorithm was used to assess quality of the studies included in the meta-analysis (Supplementary Fig. 2). The quality of evidence related to the individual outcomes analyzed was assessed using the GRADE system. (Supplementary Table 1) In addition, publication bias was assessed using funnel plots of the individual endpoints. (Supplementary Fig. 3) The baseline characteristics of the studies included are presented in Table 1. The duration of the studies ranged from 26 to 208 weeks. The dose of pioglitazone ranged from 15 to 45 mg, except for the PROFIT-J trial, where the dose was chosen based on gender. Four studies had an active control arm in the form of sulfonylurea (TOSCA.IT, PERISCOPE, Giles et al. 2008, and Giles et al. 2010), while the rest had standard of care in the control arm without pioglitazone. Two studies (J-SPIRIT and Kaneda et al.) had no anti-hyperglycemic agents in the control arm or in the pioglitazone arm as supportive medications. In J-SPIRIT, the recruited patients were diagnosed with IGT or new-onset T2DM after the detection of stroke. We analyzed data from patients with newly diagnosed T2M, with patients in the control group being managed by lifestyle modification only versus Pio. Kaku et al. used lifestyle modification as a control against Pio.

**Table 1:** Baseline characteristics of the studies included in meta-analysis.

Studies/country	Pioglitazone arm (n)	Control arm (n)	Mean age (years)	Gender % (M/F)	% HbA1c change (control group subtracted)	Pioglitazone dose (mg)	Concomitant medications (pioglitazone arm)	Concomitant medications (control arm)	Duration (weeks)
PROactive^11^(2005)/19 European countries	2605	2633	61.9	63/37	0.5	45	Metformin SU Insulin	Metformin SU Insulin	138
TOSCA.IT^12^(2017)/Italy	1535	1493	62.4	59/41	0.04	15–45	Metformin	SU	57.3
PROFIT-J^13^(2014)/Japan	254	268	69	63.2/36.8	0.53	45 (M)/30 (F)	Metformin SU AGI Insulin	Metformin SU AGI Insulin	96
Kaku et al.^14^(2009)/Japan	293	294	57.9	62.5/37.5	0.65	15–45	Metformin SU AGI Insulin	Metformin SU AGI Insulin	130–208
J-SPIRIT^15^(2015)/Japan	63	57	68.1	77.8/22.2	0.13	15–30	None	None	146
Lee et al.^16^(2013)/South Korea	60	61	60.3	71.7/28.3	0.84	15	Metformin SU Insulin	Metformin SU Insulin	52
PERISCOPE^7^(2008)/North and South America	181	179	60	68.9/31.1	0.19	15–45	Metformin Insulin	Metformin SU Insulin	78
Kaneda et al.^17^(2009)/Japan	48	48	67	75/25	0.2	15–30	None	None	26
Giles et al.^26^(2008)/USA	262	256	64.2	70.2/29.8	0.25	30–45	Insulin	SU Insulin	26
Giles et al.^27^(2010)/USA	151	149	64	56/44	0.04	15–45	Insulin	SU Insulin	52

### Macro- and microvascular outcomes

**Figure 2:**
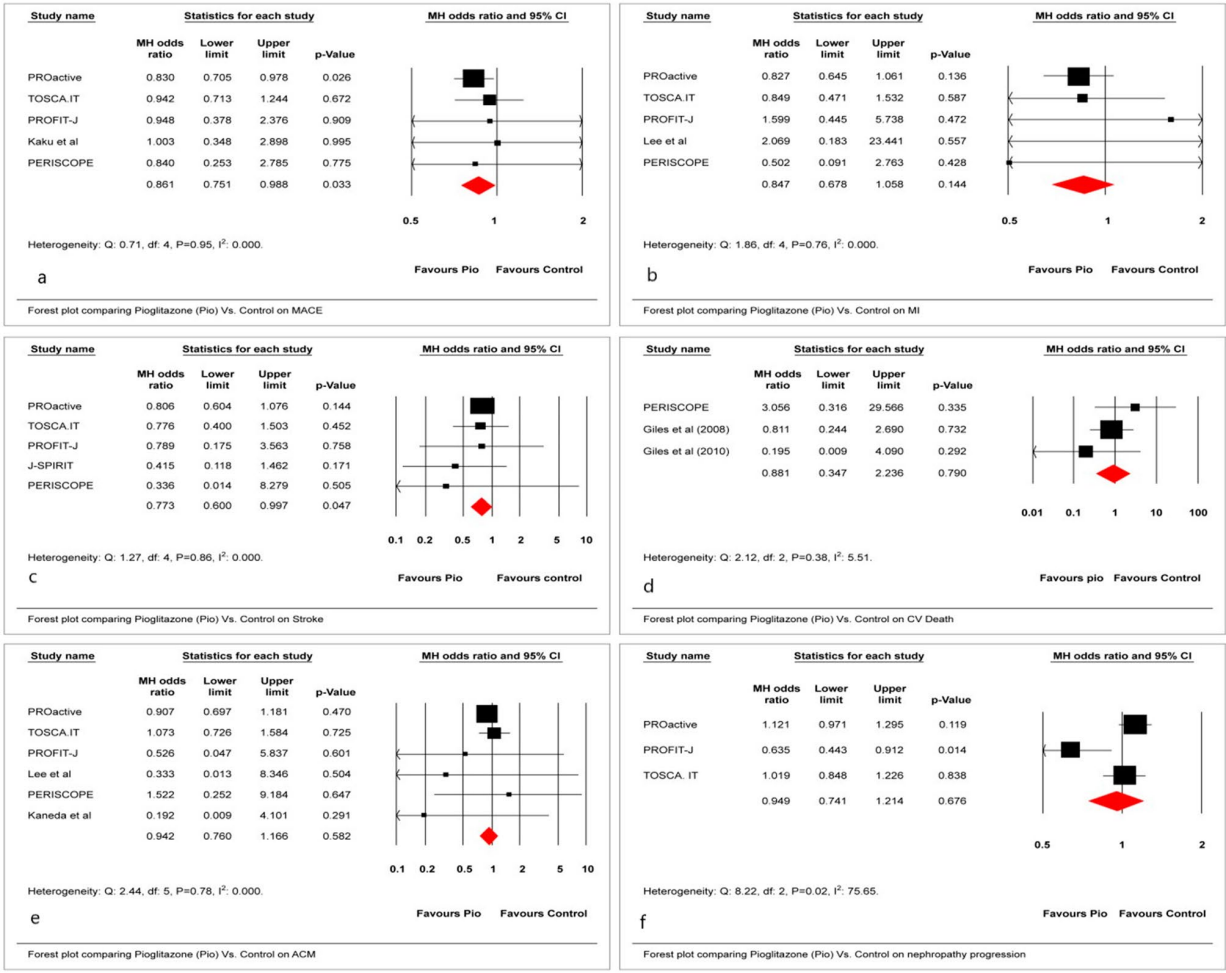
Effect of Pio vs. placebo/active control on (**a**) MACE, (**b**) MI, (**c**) stroke, (**d**) CV death, (**e**) ACM, and (**f**) nephropathy progression.

This study assessed a mixed population of patients with T2D, ranging from those with only additional CV risk factors (Kaku et al.) to those with extensive established macrovascular disease (PROactive). Compared with placebo/active control, the use of Pio resulted in 14% and 23% significant reductions in odds of MACE (MH-OR, 0.86; 95% CI 0.75–0.98) and stroke (MH-OR, 0.77; 95% CI 0.60–0.99), respectively, (Fig. 2a,c). The use of Pio was not associated with any significant effect on myocardial infarction (MI), CV death, all-cause mortality, and nephropathy progression. (Fig. 2b,d,e,f) Moreover, data related to other microvascular outcomes were not recorded in these trials. The NNT for the reduction in MACE and stroke were 80 and 151, respectively.

### Heart failure

**Figure 3:**
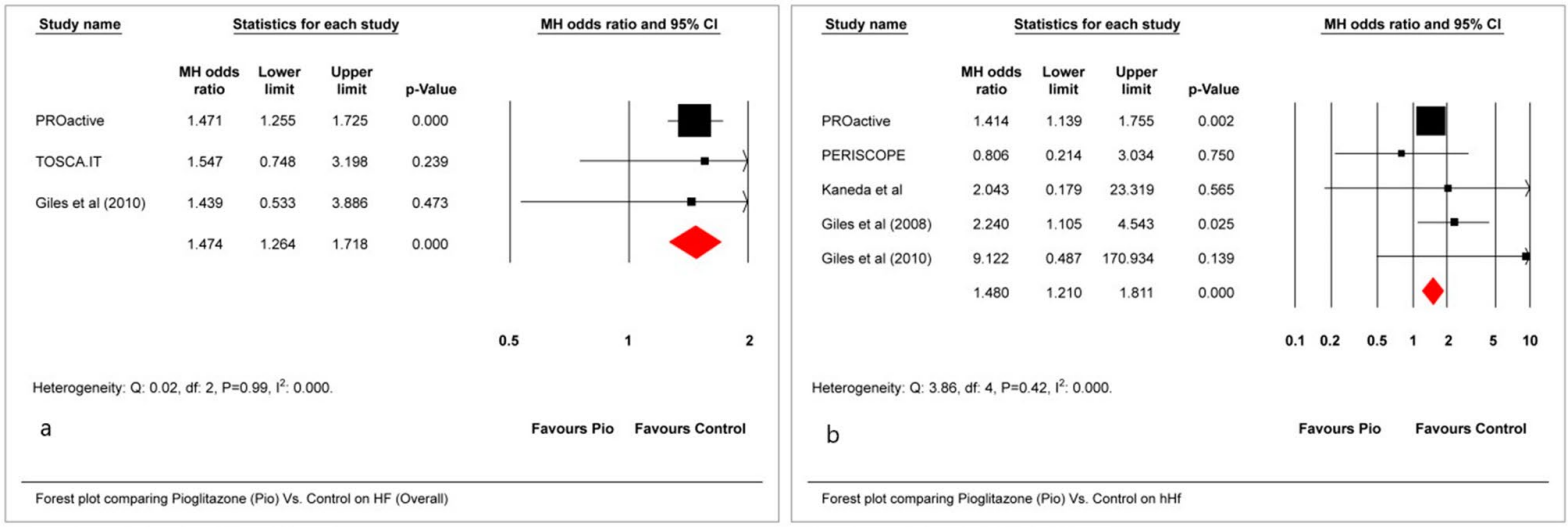
Effect of Pio vs. placebo/active control on (**a**) HF and (**b**) hHF.

A statistically significant increase in odds was noted in heart failure (HF) (MH-OR, 1.47; 95% CI 1.26–1.71) as well as hospitalization for heart failure (hHF) (MH-OR, 1.48; 95% CI 1.21–1.81) (Fig. 3a,b). The NNH for HF and hHF were 34 and 44, respectively, making these findings clinically significant.

### Other adverse outcomes

**Figure 4:**
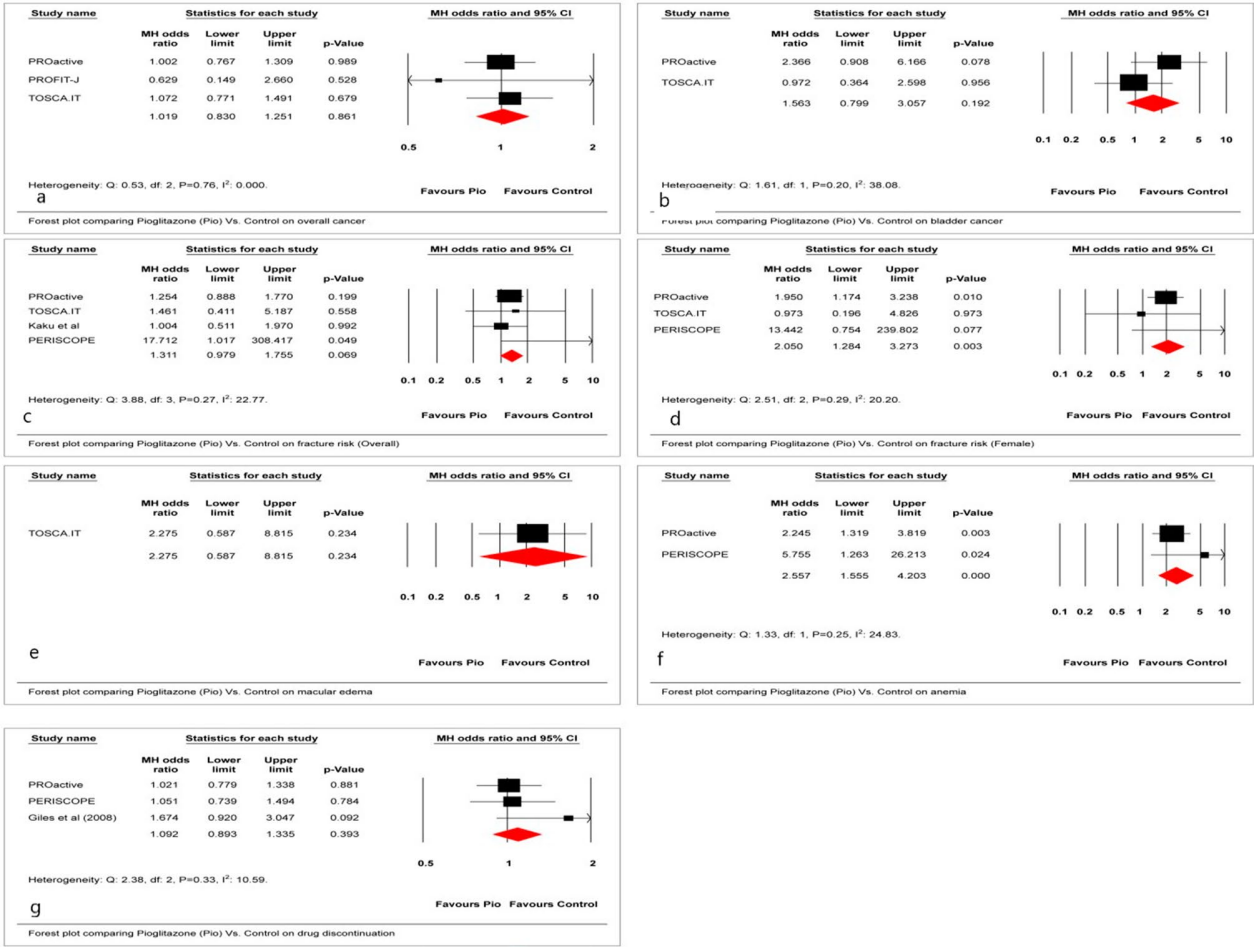
Effect of Pio vs. placebo/active control on (**a**) Overall cancer, (**b**) bladder cancer, (**c**) fracture risk (overall), (**d**) fracture risk (female), (**e**) macular edema, (**f**) anemia, and (**g**) drug discontinuation.

The overall fracture risk did not increase significantly; however, the odds of fracture risk in females was significantly increased (MH-OR, 2.05; 95% CI 1.28–3.27). (Fig. 4c,d) In addition, the odds of developing anemia was significantly increased with the Pio use (MH-OR, 2.56; 95% CI 1.55–4.21). (Fig. 4f) The impact on macular edema was not statistically significant. (Fig. 4e) Furthermore, there no significant impact on cancer in general and on bladder cancer, and the adverse effect of special interest (MH-OR, 1.56; 95% CI 0.79–3.05) (Fig. 4a,b,g). The NNH for fractures in the overall cohort and females were 197 and 637, respectively. The NNH for anemia was 87.

### Sensitivity analysis

Tests for heterogeneity revealed that there was negligible heterogeneity encountered with all parameters except nephropathy progression. Thereafter, sensitivity analysis was conducted by choosing the subgroup of studies which did not include any anti-hyperglycemic agent in the control arm (placebo). The prominent positive (MACE and stroke) and negative (hHF and fracture) outcomes were assessed for the sensitivity analysis. Fracture was subsequently excluded from the subgroup analysis in view of only a single study qualifying for inclusion. The sensitivity analysis indicated 16% lesser odds of MACE with pioglitazone compared to the non-active control arm (95% CI 0.7–0.98, p = 0.028). (Supplementary Fig. 4a) In addition, there was 12% reduced odds of developing stroke, which did not reach statistical significance (95% CI 0.59–1.03). (Supplementary Fig. 4b) There were 42% increased odds of hHF with pioglitazone in keeping with the original analysis (95% CI 1.14–1.76). (Supplementary Fig. 4c).

## Discussion

This is probably the first meta-analysis addressing specific end points studied in the CVOTs conducted on newer anti-diabetics (SGLT2i, DPP IVi, GLP1RA) as per FDA mandate of 2008^23^. A statistically significant improvement in MACE (MH-OR, 0.86; 95% CI 0.75–0.98) was noted in a heterogeneous population of patients with documented CV disease and also for those at risk for CV disease compared with both placebo and comparators (oral antidiabetic agents). This improvement in MACE was driven by a statistically significant 23% reduced odds in nonfatal strokes with Pio (MH-OR, 0.77; 95% CI 0.60–0.99). However, on subgroup analysis, though Pio continued to reduce the odds for progression to MACE, there seems to be no significant reduction in the odds for non-fatal stroke with use of Pio, in comparison to placebo. There was no effect on CV death, nonfatal MI, all-cause mortality, or progression of nephropathy. Considering the concerns about the safety of Pio, this meta-analysis also addressed the contentious issues surrounding Pio and HF, anemia, fracture, bladder cancer, and macular edema. This meta-analysis exonerates Pio from the risk of bladder cancer and macular edema. However, consistent with the previous analysis, this meta-analysis confirms a definite increased risk of HF in patients using Pio with a statistically and clinically significant increase in the odds of HF (47%) (MH-OR, 1.47; 95% CI 1.26–1.71) as well as hHF (48%) (MH-OR, 1.48; 95% CI 1.21–1.81). In addition, this increase in HF with Pio, in comparison to placebo, was also confirmed by subgroup and sensitivity analysis. Furthermore, although no overall increase in fracture risk was noted, women had a statistically significant 2.5-fold higher odds of fractures. The odds of anaemia associated with Pio use was also highly significant. (MH-OR 2.56; 95% CI 1.56–4.20).

The NNT for the reduction in MACE was 80. Though Pio seems to reduce the risk of stroke against all comparators, this benefit does not reach statistical significance when compared to placebo alone. In addition, the NNH for HF and hHF were 34 and 44, respectively. Moreover, the NNH for fractures overall and particularly in females were 197 and 637, respectively. Also, the NNH for anemia was 87.

A quick perusal of these numbers is self-explanatory; although the risk of fractures may not be significant in clinical terms, the risk of HF is a huge concern despite a definite benefit noted in patients treated with Pio on MACE. Also, the high risk of anemia is a serious concern that cannot be disregarded.

Our meta-analysis is therefore novel in that it analyzes both the statistical and clinical significance of Pio in CV outcomes for T2D, objectively weighing the pros and cons of this therapy.

In the past, multiple meta-analyses have tried to answer the question of whether Pio usage is beneficial or detrimental, with none of the studies showing any conclusive evidence. A meta-analysis of 16 retrospective studies on thiazolidinediones (TZDs) rosiglitazone was compared with Pio^20^, revealing that in T2D, use of rosiglitazone was associated with significantly higher odds of congestive heart failure, myocardial infarction, and death relative to pioglitazone in real-world settings. Of course, this meta-analysis is restricted by multiple confounding factors having studied only observational data. Another meta-analysis of 9 trials with 12,026 participants on cardiovascular outcomes of Pio in patients with T2D, pre-diabetes, and insulin resistance revealed that Pio therapy was associated with a lower risk of MACE in patients with pre-diabetes or insulin resistance (RR 0.77, 95% CI 0.64–0.93), and diabetes (RR 0.83; 95% CI 0.72–0.97). Risk of heart failure (RR 1.32; 95% CI 1.14–1.54), bone fracture (RR 1.52; 95% CI 1.17–1.99), oedema (RR; 1.63; 95% CI 1.52–1.75), and weight gain (RR 1.60; 95% CI 1.50–1.72) were found to be increased in the Pio group^20^. The findings of this meta-analysis are on similar lines as our meta-analysis, although our findings are specific to patients with T2D and not a heterogeneous cohort like prediabetes and insulin resistance, the definitions of which may vary. Furthermore, presentation of our findings as NNH and NNT renders our data more applicable clinically. Lee et al. conducted a meta-analysis of three randomized controlled trials with 4980 participants studying the use of Pio in stroke with insulin resistance, prediabetes, and diabetes mellitus, indicating that Pio therapy was associated with a lower risk of recurrent stroke (hazard ratio [HR] 0.68; 95% CI 0.50–0.92; P = 0.01) and future major vascular events (HR 0.75; 95% CI 0.64–0.87; P = 0.0001) in comparison to active controls and placebo^19^. There was no evidence of an effect on all-cause mortality and heart failure. As is evident, this meta-analysis addressed the effect of Pio on stroke alone and was not specific to T2D, including a heterogenous group of patients with prediabetes and insulin resistance. Moreover, this meta-analysis did not segregate active controls from placebo rendering their results less robust than our meta-analysis which has addressed both subgroups of comparators. Another meta-analysis of 19 trials with duration ranging from 4 months to 3.5 years with a total of 16,390 patients showed that treatment with Pio resulted in a reduction in the composite of death, myocardial infarction, or stroke against placebo or comparator oral anti-diabetics (HR, 0.82; 95% CI 0.72–0.94; P = 0.005)^22^. Serious heart failure was reported in 200 (2.3%) of the pioglitazone-treated patients and 139 (1.8%) of the control patients (HR 1.41; 95% CI 1.14–1.76; P = 0.002). The results of this analysis are restricted by their heterogeneity, as a clear assessment of CV outcomes in studies of less than 15 months would not be comparable to CV outcomes in studies over 3 years, rendering the findings of this meta-analysis debatable. Naturally, this meta-analysis published in 2007 did not include relevant data published after this. No adverse events other than HF were represented, nor was the clinical relevance by way of NNH or NNT documented.

### Study limitations

This meta-analysis has several limitations. First, there was significant heterogeneity in the baseline characteristics of the patients recruited in the trials. Some of the studies included MACE, nonfatal MI, and stroke as the outcomes of interest, while others included MACE plus (including unstable angina), total number of MI, and stroke, inclusive of deaths from these conditions. However, this heterogeneity was inevitable considering that most of these studies were conducted prior to the development of a uniform guideline by the recommending authorities like the FDA. Second, a few of the interventional trials had a shorter duration and small number of participants, which could have skewed the data. Third, instead of the individual patient data, the aggregate values were used to calculate the MH-OR. Fourth, the time-frame-based analysis on the outcomes of interest was difficult to perform because of the effect size estimation with the MH-OR and not the hazard ratio. Finally, the qualitative heterogeneity of the individual studies included must be considered prior to analyzing the results of this meta-analysis.

### Strengths of the study

This is probably the first study to assess outcomes related to those with established T2D specifically, recieveing Pio. Studies such as IRIS, which included patients with pre-diabetes^15^ have been excluded, making this meta-analysis specific to T2D in contrast to previous publications. Second, the smaller studies were not excluded to increase the analyzable numbers and ensure thereby that adverse events were ascertained with more accuracy. Third, larger studies like TOSCA-IT, which have not been included in the previous meta-analysis, were analyzed. Moreover, a pooled analysis was conducted to examine not only the possible positive outcomes of interest but also established adverse events associated with Pio, thereby enabling us to calculate both NNT and NNH, resulting in exploring the beneficial impact of Pio in the context of adverse outcomes, objectively. Fourth, the tests of heterogeneity revealed no significant impact on the effect size, except for nephropathy progression. Hence, a definitive conclusion could be drawn based on a robust primary analysis. Finally, a subgroup and sensitivity analysis were performed on the most prominent outcomes (those with significant effect sizes), and there was a very close approximation to the original effect size, except that the odds for stroke reduction with Pio, which did not reach statistical significance against placebo.

## Conclusion

The use of Pio resulted in significant reduction in MACE, while a reduction in stroke which was seen against all comparators failed to reach statistical significance on subgroup analysis when compared to placebo. There was a definite increase in HF, hHF, and anaemia, which would deter use of the drug in clinical settings, except in only very specific situations, where the patient is at high risk of developing CV disease, but newer molecules such as GLP1RA, SGLT2i, and DPP4i, which either provide robust or at least neutral outcomes with a minimal likelihood of adverse events, are contraindicated. The ADA EASD algorithm^28^ also recommends the use of Pio, where these safer molecules cannot be prescribed due to cost constraints. Hence, Pio should be prescribed by health care professionals only after thorough and detailed discussion with the patients about its adverse effects.

## Supplementary information

Supplementary Information.

## Abbreviations

Pio: Pioglitazone
T2DM: Type 2 diabetes mellitus
MACE: Major adverse cardiac events
MI: Myocardial infarction
CV: Cardiovascular
ACM: All-cause mortality
hHF: Hospitalization for heart failure
SGLT2i: Sodium glucose cotransporter-2 inhibitors
GLP1RA: Glucagon-like peptide-1 receptor analog
NNT: Number needed to treat
NNH: Number needed to harm
