# Supplementary material for: Assessing the need for pioglitazone in the treatment of patients with type 2 diabetes: a meta-analysis of its risks and benefits from prospective trials
Source: Sci Rep. 2020 Sep 25;10:15781. doi: 10.1038/s41598-020-72967-8 (PMC7519682; doi:10.1038/s41598-020-72967-8)
Supplement: Supplementary file 1 — Supplementary Information. [file 41598_2020_72967_MOESM1_ESM.pdf]

TITLE PAGE

Assessing the need for pioglitazone in the treatment of patients with type 2 diabetes: A meta-analysis of its risks and benefits from prospective trials

Binayak Sinha[1] & Samit Ghosal[2]

1. Consultant Endocrinologist: AMRI Hospitals KB 24, KB Block, Sector III, Bidhannagar, Kolkata 700098. India.

2. Consultant Endocrinologist: Nightingale Hospital. 11 Shakespeare Sarani. Kolkata 700071. India.

Corresponding author: Samit Ghosal.

E-mail address: ramdasghosal@gmail.com

Supplementary Table 1: Assessment of quality of evidence for the outcomes analyzed using the GRADE system

Outcomes of interest	Trials, n	Effect size: MH-OR; (95% CI)	P-value	I^2	Grading of Recommendations, Assessment, Development, and Evaluation
MACE	5	0.86; (0.75–0.98)	0.03	0.000	⊕⊕⊕⊕
MI	5	0.85; (0.68–1.06)	0.14	0.000	⊕⊕⊕ϴ
Stroke	5	0.77; (0.60–0.99)	0.04	0.000	⊕⊕⊕ϴ
CV death	3	0.88; (0.35–2.24)	0.79	5.51	⊕⊕⊕ϴ
ACM	6	0.94; (0.76–1.16)	0.58	0.000	⊕⊕⊕⊕
Nephropathy progression	3	0.95; (0.74–1.21)	0.68	75.65	⊕⊕⊕ϴ
hHF	5	1.47; (1.26–1.71)	<0.001	<0.001	⊕⊕⊕ϴ
HF	3	1.48; (1.21–1.81)	<0.001	<0.001	⊕⊕ϴ
Cancer	3	1.02; (0.83-1.25)	0.86	0.000	⊕⊕ϴϴ
Fracture	4	1.31; (0.98-1.76)	0.06	22.77	⊕⊕ϴϴ
Macular edema	1	2.28; (0.59-8.82)	0.23	NA	⊕ϴϴϴ
Anaemia	2	2.56; (1.56-4.20)	<0.001	24.83	⊕ϴϴϴ
Drug discontinuation	3	1.09; (0.89-1.34)	0.38	10.59	⊕ϴϴϴ

Supplementary figure 1: Web search strategy

Search Name: Pio: Cochrane Library (Access provided by Royal College of Physicians)

Date Run: 12/08/2018 17:43:52

Comment:

ID	Search	Hits
#1	MeSH descriptor: [Diabetes Mellitus, Type 2] explode all trees	17440
#2	MeSH descriptor: [Thiazolidinediones] explode all trees	1811
#3	MeSH descriptor: [Pioglitazone] explode all trees	1053
#4	#2 OR #3	1811
#5	#1 OR #2 OR #3 in Trials	18087
#6	#1 AND #4	1083
#7	(major adverse cardiac events):ti,ab,kw (Word variations have been searched)	5899
#8	(MACE):ti,ab,kw (Word variations have been searched)	2957
#9	(myocardial infarction):ti,ab,kw (Word variations have been searched)	30757
#10	(stroke):ti,ab,kw (Word variations have been searched)	54538
#11	(cardiovascular death):ti,ab,kw (Word variations have been searched)	12329
#12	(all-cause mortality):ti,ab,kw (Word variations have been searched)	9388
#13	(heart failure):ti,ab,kw (Word variations have been searched)	35467
#14	(hospitalization for heart failure):ti,ab,kw (Word variations have been searched)	10194
#15	(microvascular outcomes):ti,ab,kw (Word variations have been searched)	1384
#16	#7 OR #8 OR #9 OR #10 OR #11 OR #12 OR #13 OR #14 OR #15	113261
#17	#16 AND #5	1410
#18	#16 AND #6	127

Supplementary Figure 2: Quality of selected studies using the Cochrane risk of bias algorithm

	Random sequence generation	Allocation concealment	Blinding of participants and personnel	Blinding of outcome assessment	Incomplete outcome data	Selective reporting	Other bias
PROactive[11] 2005	+	+	+	+	+	+	+
TOSCA.IT[12] 2017	+	+	+	+	+	+	+
PROFIT-J[13] 2014	?	?	-	-	+	+	+
Kaku et al[14] 2009	?	?	-	+	+	?	+
J-SPIRIT[15] 2017	?	?	-	?	+	?	?
Lee et al[16] 2013	?	?	?	?	+	?	?
PERISCOPE[7] 2008	+	+	+	+	+	+	+
Kaneda et al[17] 2009	?	?	+	-	+	?	+
Giles et al[26]2008	?	+	?	?	+	-	?
Giles et al[27] 2010	?	?	+	?	+	?	?

+ Low risk of bias

-High risk of bias

? Unclear risk of bias

Supplementary figure 3: Funnel plots assessing publication bias (a) MACE, (b) Stroke, (c) Fracture risk, and (d) hHF.

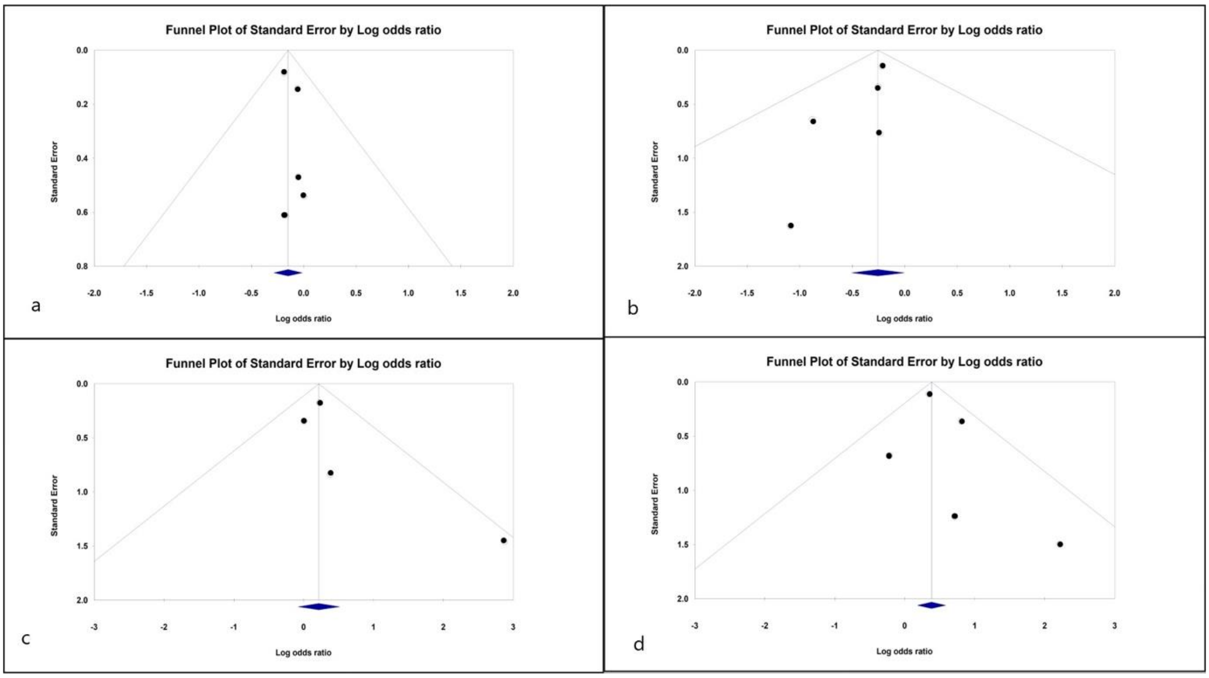

Supplementary Figure 4: Sensitivity analysis comparing Pioglitazone versus non-active control on (a). MACE, (b). Stroke, and (c). hHF.

Study name	Subgroup within study	Odds ratio	Lower limit	Upper limit	p-Value
PROactive	MACE	0.830	0.705	0.978	0.026
PROFIT-J	MACE	0.948	0.378	2.377	0.909
Kaku et al	MACE	1.003	0.348	2.894	0.996
		0.837	0.714	0.981	0.028

Heterogeneity: Q=0.193, df=2, I^2=0.000. P=0.91.

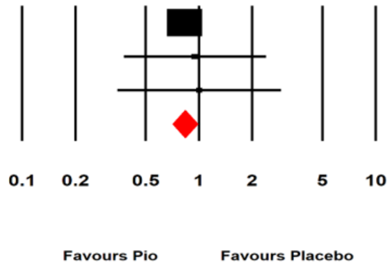

a

Study name	Subgroup within study	Odds ratio	Lower limit	Upper limit	p-Value
PROactive	STROKE	0.806	0.604	1.076	0.143
PROFIT-J	STROKE	0.789	0.175	3.560	0.758
J-SPIRIT	STROKE	0.415	0.118	1.461	0.171
		0.780	0.592	1.029	0.078

Heterogeneity: Q=1.016, df=2, I^2=0.000. P=0.60.

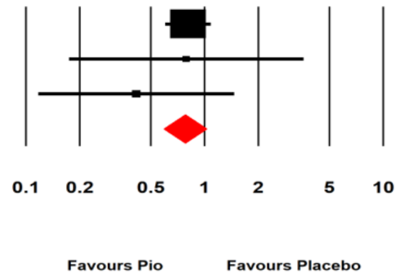

b

Study name	Subgroup within study	Odds ratio	Lower limit	Upper limit	p-Value
PROactive	HHF	1.414	1.139	1.755	0.002
Kaneda et al	HHF	2.043	0.179	23.318	0.565
		1.418	1.143	1.759	0.001

Hetrogeneity: Q=0.087, df=1, I^2=0.000. P=0.77.

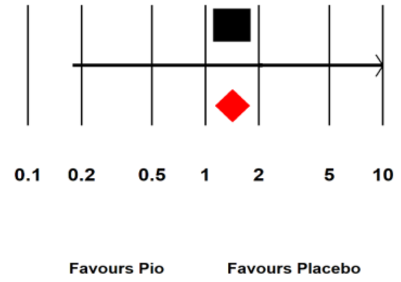

c
